# Combination chemoimmunotherapy with monthly cycles of 5-fluorouracil + interferon-alfa-2b in previously-treated advanced cancer

**DOI:** 10.1186/2051-1426-1-S1-P85

**Published:** 2013-11-07

**Authors:** Walter Quan, Vivek Khemka, Laura S Martell, Marci J Pierog

**Affiliations:** 1 Western Regional Medical Center, Goodyear, AZ, USA

## 

The in vitro antineoplastic activity of the combination of 5-fluorouracil (5-FU) and interferon-alfa (IFN) has been attributed to multiple mechanisms including enhanced thymidylate synthase inhibition (Elias) and increased susceptibility to interferon-stimulated natural killer cells (Imai). Our group has previously examined weekly dosing schedules of 5-FU and IFN (Quan). Monthly bolus 5-FU + interferon-alfa-2b has not undergone formal phase II testing. In our current study, 18 patients with metastatic cancer who had experienced disease progression on at least 2 prior lines of systemic therapy, have been treated with 5-FU 500 mg/M2 intravenously over 3-5 minutes followed immediately by IFN-2b 3 million IU subcutaneously daily for 3 days monthly on an outpatient basis. Patient characteristics: 12 males/6 females, median age-57 (range: 35-69), median ECOG performance status-1; most common tumor types: colon (6), adenocarcinoma of the lung (4), rectum (3), pancreas (3); common metastatic sites: liver (11), lymph nodes (10), lungs (9). Median number of prior chemotherapy regimens: 3 (range: 2-8). Most common toxicities: rigors, nausea/emesis, fever, and fatigue. Median number of cycles received: 2 (1-11). Sixteen patients are evaluable for response (2 are too early). Two ongoing partial responses have been seen: One patient with adenocarcinoma of the lung (lung + lymph node metastases) at 8.9+ months and one with colon (liver and lymph node metastases at 1.25+ months. Thymidylate synthase levels are being analyzed. Monthly bolus 5-FU followed by subcutaneous IFN-2b is well-tolerated in previously-treated metastatic cancer and shows some evidence of activity in adenocarcinoma of the lung and colon cancer.

